# Prognostic implications of ezrin and phosphorylated ezrin expression in non-small cell lung cancer

**DOI:** 10.1186/1471-2407-14-191

**Published:** 2014-03-15

**Authors:** Tiefeng Jin, Jingchun Jin, Xiangyu Li, Songnan Zhang, Yun Ho Choi, Yingshi Piao, Xionghu Shen, Zhenhua Lin

**Affiliations:** 1 Department of Pathology & Cancer Research Center, Yanbian University Medical College, Yanji 133002, China; 2 Department of Internal Medicine, Yanbian University Hospital, Yanji 133000, China; 3 Department of Oncology, Yanbian University Hospital, Yanji 133000, China; 4 Department of Anatomy, Medical School, Institute for Medical Sciences, Chonbuk National University, Jeonju 561-756, Republic of Korea

**Keywords:** Lung cancer, Ezrin, Phosphorylated ezrin, Immunohistochemistry, Survival analysis

## Abstract

**Background:**

The cytoskeletal organizer ezrin is a member of the ezrin-radixin-moesin (ERM) family and plays important roles in not only cell motility, cell adhesion, and apoptosis, but also in various cell signaling pathways. Phosphorylation at Thr-567 and Tyr-353 are key regulatory events in the transition of the dormant to active form of ezrin. This study investigated the prognostic implications of ezrin and phosphorylated ezrin (p-ezrin) expression in non-small cell lung carcinoma (NSCLC).

**Methods:**

Ezrin and p-ezrin protein expressions were examined by immunohistochemistry in 150 NSCLC and adjacent non-tumor tissues and 14 normal lung tissues. qRT-PCR was used to determine ezrin mRNA expression levels in fresh tissues. The correlations between overexpression of ezrin and p-ezrin and the clinicopathological features of NSCLC were analyzed. The survival rates were calculated by the Kaplan-Meier method for 108 NSCLC cases.

**Results:**

Ezrin and ezrin^Thr-567^ proteins showed cytosolic and membranous staining patterns; however, ezrin^Tyr-353^ protein only showed cytosolic staining. Ezrin and p-ezrin were significantly upregulated in NSCLC compared with the normal counterparts. Increased ezrin, ezrin^Thr-567^, and ezrin^Tyr-353^ levels were correlated with the late stage and poor differentiation of NSCLC. However, only ezrin^Thr-567^ was correlated with the presence of lymph node metastasis. In regard to survival, only ezrin^Thr-567^ was related with the overall survival time of patients with NSCLC, and both ezrin and ezrin^Thr-567^ were associated with shortened survival time for patients with early stage NSCLC.

**Conclusions:**

Ezrin and p-ezrin, especially ezrin^Thr-567^, may prove to be useful as a novel prognostic biomarker of NSCLC.

## Background

Lung cancer is a leading cause of cancer-related death worldwide, with over one million cases diagnosed each year [1]. Approximately 85% of lung cancers are non-small cell lung cancer (NSCLC) [2]. Molecular target therapy is one of the promising field of NSCLC treatment, and its target includes EGFR (epidermal growth factor receptor), EML4-ALK (echinoderm microtubule associated protein like4-anaplastic lymphoma kinase). EGFR TKI (gefitinib and erlotinib) and EML4/ALK inhibitor (Crizotinib) have achieved better results in the clinical therapy of advanced NSCLC [3,4]. Despite progress in the multimodality treatment of lung cancer, prognosis is still poor, with 10–15% 5-year survival rates. More than 90% of deaths from NSCLC are attributable to metastases [5].

The cytoskeletal organizer ezrin was first identified as an important metastatic regulator in rhabdomyosarcoma and osteosarcoma [6,7]. Ezrin is a member of the ezrin-radixin-moesin (ERM) family and acts as a cross-linker between the plasma membrane and the actin cytoskeleton [8]. In its inactive form, ezrin is located in the cytoplasm and its C-terminal domain, an F-actin-binding site, is masked by the N-terminal domain of ezrin or other ERM family member proteins. Once ezrin is activated by threonine and tyrosine phosphorylation, it assumes an active form, in which its N-terminal domain binds the cell membrane and its C-terminal domain binds to F-actin [8,9]. Ezrin plays important roles not only in cell motility, cell adhesion, and apoptosis, but also in various cell signaling pathways. Ezrin is synthesized in a dormant form in which the N-terminal domain in bound to the C-terminal domain, thus mutually blocking their capacity to bind other molecules [10].

Phosphatidylinositol 4, 5-bisphosphate (PIP2) binding and the phosphorylation of threonine 567 (Thr-567) and tyrosine 353 (Tyr-353) in ezrin are involved in the switching of ezrin from the dormant form to its active state. Once phosphorylated, ezrin binds more tightly to the membrane. Phosphorylated ezrin (p-ezrin) is often associated with the stimulation of cellular functions. Phosphorylation of Tyr-353 in ezrin is regulated through the PI3-kinase/Akt pathway, and phosphorylation of Thr-567 depends on the p38 MAP-kinase activity, and these phosphorylation events promote tumor amplification, metastasis and invasion. Phosphorylation of Thr-567 and Tyr-353 are key events that regulate the transition from the dormant state of ezrin to its active form [11]. These findings suggest that phosphorylated ezrin might be a potential molecular target for cancer therapy.

A few studies to date have reported an association between ezrin/p-ezrin expression and clinicopathological parameters, as well as its prognostic role in lung cancer. Thus, we analyzed the expression and localization of ezrin/p-ezrin in NSCLC compared with the normal counterparts, determined its relationship with clinicopathological parameters, and investigated its prognostic value for NSCLC patients based on tumor stage and survival data. We found that ezrin and p-ezrin (ezrin^Thr-567^ and ezrin^Tyr-353^) are frequently upregulated in NSCLC compared with the normal counterparts, and are related with the poor differentiation and late clinical stage of NSCLC. However, only ezrin^Thr-567^ was related with the presence of lymph node metastasis and the overall survival time of NSCLC patients, indicating that ezrin, especially ezrin^Thr-567^, may prove to be useful as a novel prognostic biomarker of NSCLC.

## Methods

### Ethics statement

This study complied with the Helsinki Declaration and was approved by the Human Ethics and Research Ethics committees of Yanbian University Medical College in China. Through the surgery consent form, patients were informed that resected specimens were stored by the hospital and potentially used for scientific research, and that their privacy would be maintained. Follow-up survival data were collected retrospectively through medical record analyses.

### Clinical samples

A total of 150 NSCLC tissue microarray samples, including 82 cases of lung adenocarcinoma and 68 cases of lung squamous cell carcinomas (SCCs), were collected from Shanghai Outdo Biotech Co. Ltd. between Dec. 2004 and Jan. 2008. (Outdo Biotech) and Affiliated Hospital of Chengde Medical College. All cases of NSCLC used in this study were primary tumor, and were not treated before surgery. Fourteen cases of normal lung tissue were obtained from autopsy samples in Yanbian University Medical College. All tissues were routinely fixed in 10% buffered formalin and embedded in paraffin blocks.

The pathological parameters, including age, gender, tumor size, clinical stage, differentiation and the presence of nodal metastasis, were carefully reviewed in all 150 samples. The patients’ age ranged from 36 to 78 years, with a mean age of 60.2 years. The male to female ratio was 112:38. TNM staging was assessed according to the staging system established by the American Joint Committee on Cancer (AJCC 7^th^ edition). Of the 150 NSCLC cases, 54 cases were TNM stage I (TNM stage IA = 14, TNM stage IB = 40), 44 cases were TNM stage II (TNM stage IIA = 35, TNM stage IIB = 9), 45 cases were TNM stage III (TNM stage IIIA = 34, TNM stage IIIB = 11), and 7 cases were TNM stage IV. Thirty-four cases were well differentiated, 89 cases were moderately differentiated, and 27 cases were poorly differentiated. Of the 150 NSCLC samples, 96 cases were lymph node (LN) metastasis-negative, and 54 cases were LN metastasis-positive. A total 108 of NSCLC patients had follow-up records for more than 5 years, and the follow-up deadline was March 2012. By March 2012, 54 patients had died while 54 patients remained alive. The median survival time was 43.2 months. The survival time was counted from the date of surgery to the follow-up deadline, or date of death (all of them died of cancer recurrence or metastasis).

### Immunohistochemistry for ezrin and p-ezrin in paraffin-embedded tissues

The Dako LSAB kit (Dako, Glostrup, Denmark) was used for immunohistochemistry. Serial 4 μm-thick tissue sections were prepared on silane-coated slides (Sigma, St. Louis, MO, USA), and deparaffinized, rehydrated and incubated with 3% H_2_O_2_ in methanol for 10 min at room temperature to eliminate endogenous peroxidase activity. The antigen was retrieved at 95°C for 20 min by placing the slides in 10 mM sodium citrate buffer (pH 6.0). The slides were then incubated with primary antibodies against ezrin (1:50, #3145; anti-rabbit polyclonal antibody, Cell Signaling Technology, Boston, USA), ezrin^Tyr-353^ (1:150, #11063, anti-rabbit polyclonal antibody, Signalway Technology, Maryland, USA), and ezrin^Thr-567^, (1:150, #11202, anti-rabbit polyclonal antibody, Signalway Technology) at 4°C overnight. After incubation at room temperature for 30 min with biotinylated secondary antibody, the slides were incubated with streptavidin-peroxidase complex at room temperature for 30 min. Immunostaining was developed using chromogen, 3,3′-diaminobenzidine, and counterstained with Mayer’s hematoxylin. Rabbit IgG isotope was used as control and the results were negative. Positive tissue sections were processed without primary antibody as negative controls.

### Evaluation of immunohistochemical staining

All slides were evaluated independently by two pathologists without knowledge of clinical outcome. The interpretation criteria were previously described by Elzagheid A et al. [12] and Lin L et al. [13]. Combined the staining intensity, the immunostaining for ezrin/p-ezrin was mainly semi-quantitatively scored as ‘-’ (negative, no or less than 5% positive cells), ‘+’ (5–25% positive cells), ‘++’ (26–50% positive cells) and ‘+++’ (more than 50% positive cells). The ‘strongly positive’ descriptor was used to describe ‘++’ and ‘+++’ scored cells. For survival analysis, ezrin/p-ezrin expression level was denoted as high expression (‘++’ and ‘+++’) or low expression (‘-’ and ‘+’).

### Quantitative real-time polymerase chain reaction (qRT-PCR)

Total RNA was extracted using Trizol reagent (Invitrogen, Carlsbad, CA) from 21 NSCLC fresh tissue samples, 15 adjacent lung tissues and 8 normal tissue counterparts. First-strand cDNA was synthesized by PrimeScript reverse transcriptase (TaKaRa Bio, Dalian, China) and oligo (dT) following the manufacturer’s instructions. Real-time PCR was performed using double-stranded DNA-specific SYBR Premix Ex TaqTM II Kit (TaKaRa Bio) on a Bio-Rad sequence detection system according to the manufacturer’s instructions. Double-stranded DNA specific expression was tested by the comparative Ct method using 2^-ΔΔCt^. Ezrin primers were as follows: 5′-TGGAGTTGATGCCCTTGGAC-3′ and 5′-AGTCAGGTGCCTTCTTGTCG-3′. GAPDH primers were as follows: 5′-CATCACCATCTTCCAGGAGCG-3′ and 5′-TGACCTTGCCCACAGCCTTG-3′. All assays were performed in triplicate at least three times.

### Statistical analysis

Statistical analysis was performed using the Chi-square (*x*^*2*^-test) test, Mann–Whitney test and Kaplan–Meier test and the SPSS software program for Windows, version 17.0 (SPSS, Chicago, USA). A *P*-value less than 0.05 was considered as statistically significant.

## Results

### Quantification of ezrin, ezrin^Thr-567^ and ezrin^Tyr-353^ overexpression in NSCLC by immunohistochemistry and qRT-PCR

**Figure 1:**
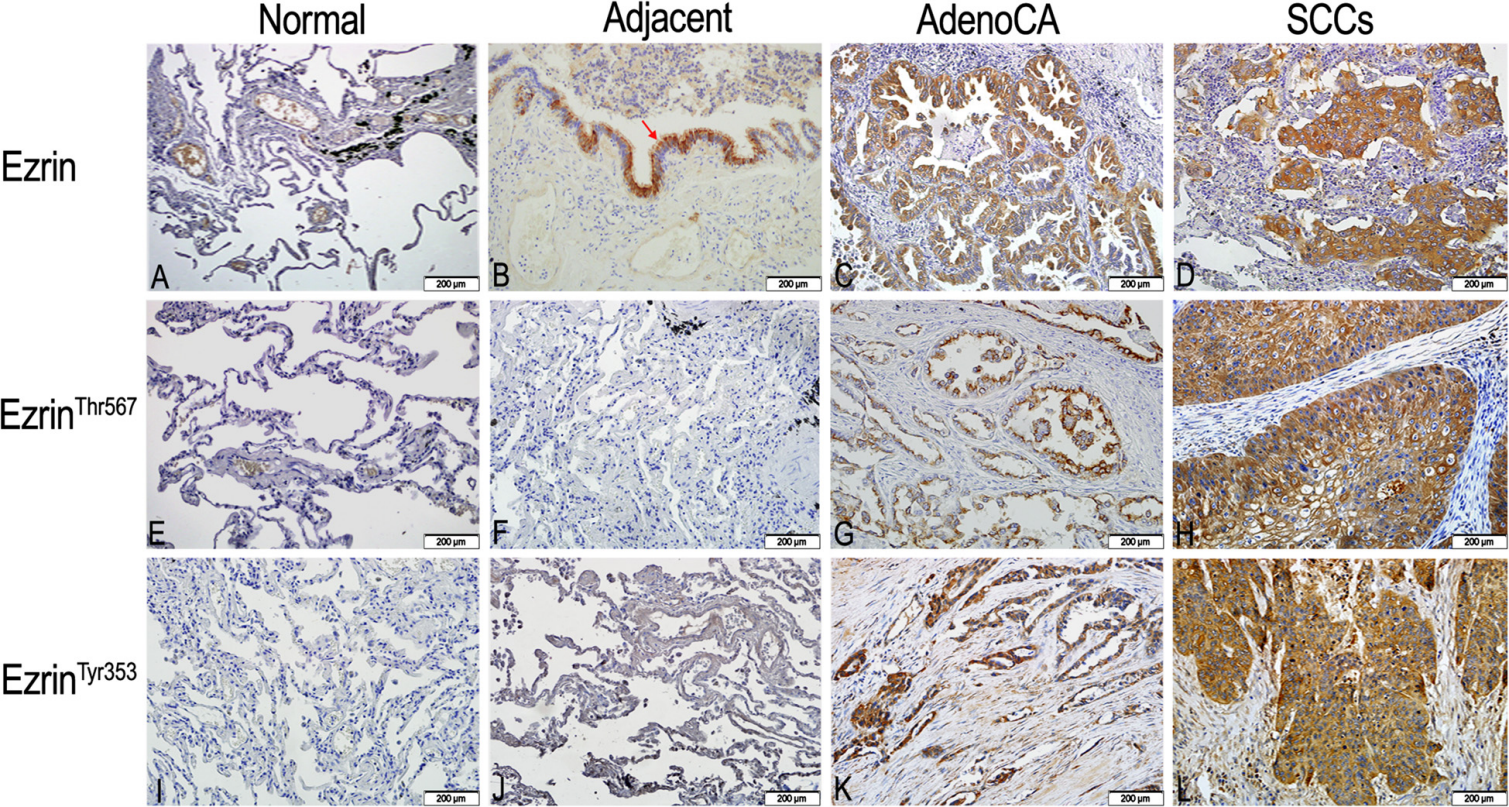
**Ezrin and p-ezrin protein expression in NSCLC and normal counterparts. (A)** Negative ezrin protein expression in normal lung tissues. **(B)** Positive ezrin expression in the cytoplasm of normal columnar epithelia (*red arrows*) of terminal bronchiole in adjacent non-tumor tissues. **(C)** Positive ezrin expression in the cytoplasm of adenocarcinoma cells of lung. **(D)** Strong positive expression of ezrin protein in the cytoplasm of squamous cell carcinomas (SCCs). **(E, F)** Negative ezrin^Thr567^ expression in normal lung tissues and adjacent non-tumor tissues. **(G)** Strong positive expression of ezrin^Thr567^ in the cytoplasm of lung adenocarcinoma. The positive signals were concentrated in the perinucleus. **(H)** Strong positive expression of ezrin^Thr567^ in the cytoplasm and membranes of lung SCCs. **(I, J)** Negative ezrin^Thr353^ expression in normal lung tissues and adjacent non-tumor tissues. **(K)** Strong expression of ezrin^Tyr353^ in the cytoplasm of lung adenocarcinomas. **(L)** Strong expression of ezrin^Tyr353^ in the cytoplasm of lung SCCs. Magnification 200× in **A–L**.

We first performed immunohistochemistry for ezrin, ezrin^Thr-567^ and ezrin^Tyr-353^ in 150 samples of paraffin-embedded NSCLC samples, 150 adjacent lung tissues and 14 normal tissue counterparts. Ezrin and ezrin^Tyr-353^ showed mainly cytoplamic staining, while ezrin^Thr-567^ showed cytosolic and membranous staining patterns in NSCLC samples. Ezrin and p-ezrin (both Thr-567 and Tyr-353) proteins showed significantly higher levels in NSCLC samples compared with adjacent non-tumor and normal lung tissues. The percentages of positive ezrin, ezrin^Thr-567^ and ezrin^Tyr-353^ cells in adjacent non-tumor tissues were 31.3%, 14.0% and 11.3%, respectively, and 35.7%, 14.3% and 7.1% in normal lung tissue counterparts, respectively. However, the rates of positive ezrin, ezrin^Thr-567^ and ezrin^Tyr-353^ expression were significantly higher in NSCLC than in the adjacent non-tumor tissues and normal lung tissue counterparts, with rates of 62.7%, 63.3% and 71.3% in NSCLC, respectively (*P* < 0.01). The percentages of strongly positive ezrin, ezrin^Thr-567^ and ezrin^Tyr-353^ cells were 40.7%, 45.3% and 48.0% in NSCLC, respectively, and were also significantly higher than in adjacent non-tumor tissues and normal tissue counterparts (*P* < 0.01). In contrast, in adjacent non-tumor tissues, the percentages of cells with strongly positive ezrin, ezrin^Thr-567^ and ezrin^Tyr-353^ expression were 3.3%, 4.7% and 1.3%, respectively, and completely negative in normal lung tissue counterparts (Table 1 and Figure 1).

**Table 1:** **Expression of ezrin, ezrin**^**Thr-567 **^**and ezrin**^**Tyr-353 **^**in NSCLC**

		**Ezrin**		**Ezrin**^**Thr-567**^		**Ezrin**^**Tyr-353**^	
**Diagnosis**	**No. of cases**	**Positive rate**	**Strongly positive rate**	**Positive rate**	**Strongly positive rate**	**Positive rate**	**Strongly positive rate**
		**(+ ~ +++, %)**	**(++ ~ +++, %)**	**(+ ~ +++, %)**	**(++ ~ +++, %)**	**(+ ~ +++, %)**	**(++ ~ +++, %)**
NSCLC	150	94 (62.7%)**	61 (40.7%)**	95 (63.3%)**	68 (45.3%)**	107 (71.3%)**	72 (48.0%)**
Adjacent non-tumor	150	47 (31.3%)	5 (3.3%)	21 (14.0%)	7 (4.7%)	17 (11.3%)	2 (1.3%)
Normal lung tissues	14	5 (35.7%)	0 (0.0%)	2 (14.3%)	0 (0.0%)	1 (7.1%)	0 (0.0%)

Positive rate: percentage of positive cases with ‘+’, ‘++’, and ‘+++’, Strongly positive rate: percentage of positive cases with ‘++’, and ‘+++’, **P < 0.01: NSCLC *vs* Adjacent non-tumor and Normal lung tissues.

**Figure 2:**
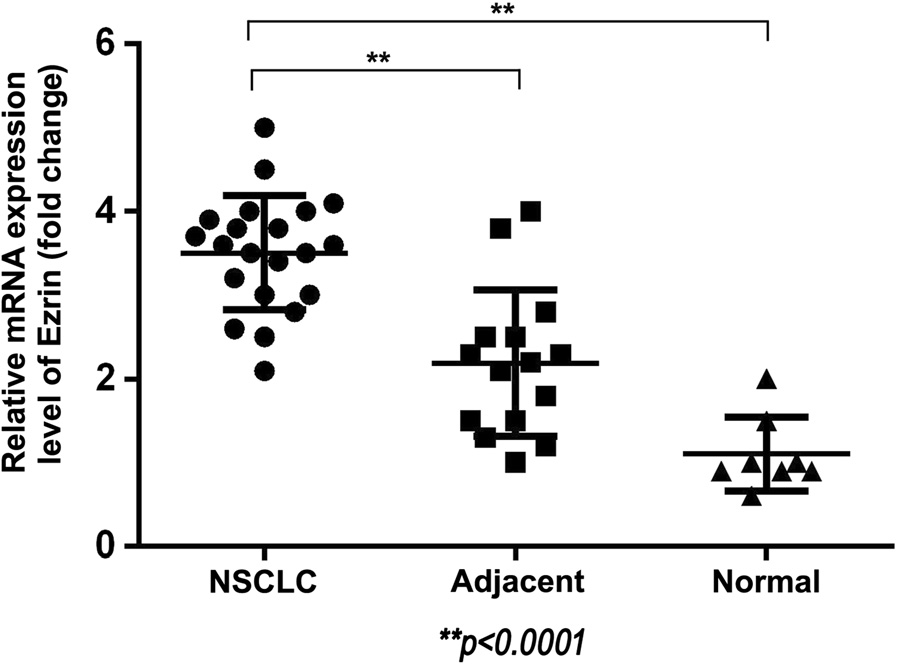
**Ezrin mRNA expression in NSCLC and normal counterparts.** qRT-PCR analysis of ezrin mRNA in NSCLC, adjacent and normal tissues. Ezrin mRNA expression levels were significantly higher in NSCLCs compared with the adjacent non-tumor and normal lung fresh tissues. Experiments were performed in triplicate for each case.

qRT-PCR data confirmed increased levels of ezrin mRNA expression in NSCLC samples compared with adjacent non-tumor tissues and normal tissue counterparts in fresh tissues (Figure 2).

### Association between overexpression of ezrin, ezrin^Thr-567^ and ezrin^Tyr-353^ proteins and clinical parameters of NSCLC

Ezrin, ezrin^Thr-567^ and ezrin^Tyr-353^ overexpression significantly correlated with the poor differentiation and late clinical stage of NSCLC. The percentages of positive ezrin, ezrin^Thr-567^ and ezrin^Tyr-353^ cells were significantly higher in poorly differentiated NSCLC cases (85.2%, 88.9% and 92.6%, respectively) compared with well differentiated NSCLC (50.0%, 47.1% and 61.8%, respectively) and moderately differentiated NSCLC cases (60.7%, 61.8% and 68.5%, respectively) (*P* < 0.01). For the TNM clinical stage, the percentages of positive ezrin, ezrin^Thr-567^ and ezrin^Tyr-353^ cells in advanced stage (III-IV) of NSCLC were 82.7%, 75.0% and 92.3%, respectively. These levels were much higher than in cases of early stage (I–II) (52.0%, 57.1% and 60.2%, respectively) (*P* < 0.01, *P* < 0.05 and *P* < 0.01, respectively). Interestingly, only ezrin^Thr-567^ overexpression was significantly correlated with the presence of LN metastasis of NSCLC. The percent of positive ezrin^Thr-567^ cells in NSCLC with LN metastasis was 79.6% (43/54), and this was statistically higher than in cases without LN metastasis (54.2%, 52/96) (*P* < 0.01), indicating that ezrin^Thr-567^ might be more accurate than ezrin or ezrin^Tyr-353^ as a marker for poor prognosis for NSCLC. Additionally, the expression status of ezrin, ezrin^Thr-567^ and ezrin^Tyr-353^ proteins was not correlated with age, gender or tumor size of patients with NSCLC (Table 2).

**Table 2:** **Correlation between overexpression of ezrin, ezrin**^**Thr-567 **^**and ezrin**^**Tyr-353 **^**proteins and clinical parameters of NSCLC**

		**Ezrin**		**Ezrin**^**Thr-567**^		**Ezrin**^**Tyr-353**^	
**Parameters**	***No. of cases***	***n *** **(%)**	***P***	***n *** **(%)**	***P***	***n *** **(%)**	***P***
**Gender**			*0.062*		*0.45*		*0.230*
Male	112	75 (67.0)		69 (61.6)		77 (68.8)	
Female	38	19 (50.0)		26 (68.4)		30 (78.9)	
**Age**			*0.077*		*0.642*		*0.364*
≧60	99	67 (67.7)		64 (64.6)		73 (73.7)	
<60	51	27 (52.9)		31 (60.8)		34 (66.7)	
**Tumor size**			*0.283*		*.791*		*0.620*
T1-2	119	72 (60.5)		76 (63.9)		86 (72.3)	
T3-4	31	22 (71.0)		19 (61.3)		21 (67.7)	
**Stage**			0.000**		.031*		0.000**
I-II	98	51 (52.0)		56 (57.1)		59 (60.2)	
III-IV	52	43 (82.7)		39 (75.0)		48 (92.3)	
**Differentiation**			*0.007*^a^**		*0.002*^a^**		*0.006*^a^**
Well	34	17 (50.0)		16 (47.1)		21 (61.8)	
Moderate	89	54 (60.7)		55 (61.8)		61 (68.5)	
Poor	27	23 (85.2)		24 (88.9)		25 (92.6)	
**LN metastasis**			*0.318*		0.002**		*0.578*
Negative	96	63 (65.6)		52 (54.2)		67 (69.8)	
Positive	54	31 (57.4)		43 (79.6)		40 (74.1)	

*P < 0.05; **P < 0.01; *a:* Poorly *vs* Well and moderately differentiated tumors.

### Evaluation of ezrin and p-ezrin as a potential prognostic marker for NSCLC by Kaplan–Meier test

**Figure 3:**
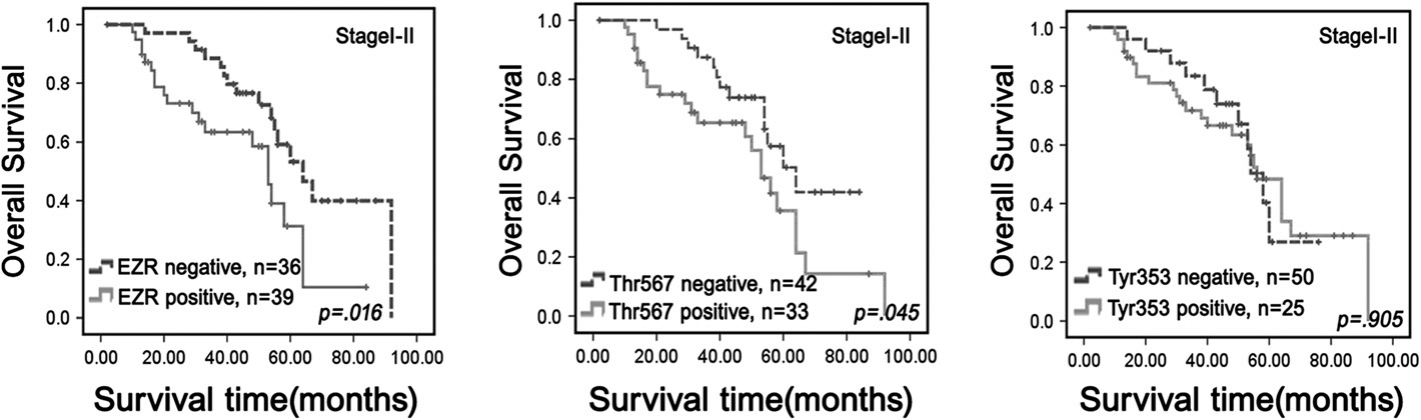
**Kaplan-Meier survival curves illustrating the significance of ezrin and p-ezrin expressions in NSCLCs.** NSCLC patients with high ezrin^Thr567^ levels had a lower overall survival rate compared with NSCLC patients with low ezrin^Thr567^ levels (log-rank *P* = 0.019). However, both ezrin and ezrin^Tyr353^ were not related with the overall survival of patients with NSCLC (log-rank *P* = 0.076, log-rank *P* = 0.093, respectively).

A total 108 of NSCLC patients were identified for analysis of prognostic evaluation. The data showed that elevated ezrin^Thr-567^ was significantly related with shorter survival times (*P* = 0.019, log-rank). However, ezrin and ezrin^Tyr-353^ expression statuses were not related with the overall survival times of patients with NSCLC (*P* = 0.076 and *P* = 0.093, respectively, log-rank) (Figure 3).

**Figure 4:**
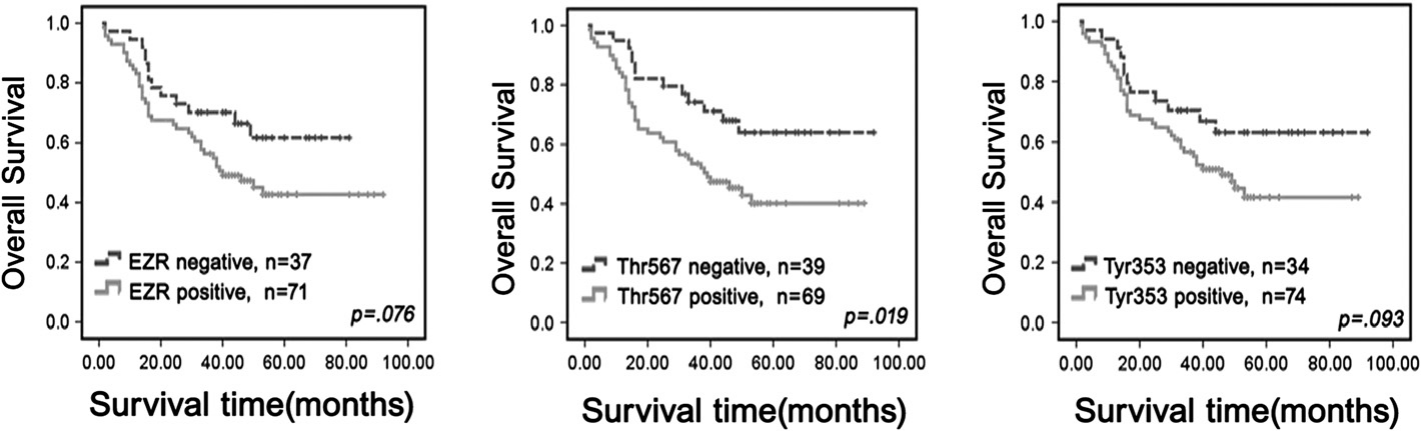
**Kaplan-Meier survival curves illustrating the significance of ezrin and p-ezrin expression in early-stage NSCLCs.** In early-stage NSCLCs (stages I–II, n = 98), patients with high ezrin and ezrin^Thr567^ levels had significantly reduced NSCLC-specific overall survival rates relative to those with low ezrin and ezrin^Thr567^ levels (log-rank *P* = 0.030), respectively. However, ezrin^Tyr353^ was not related with the survival of patients with early-stage NSCLC.

In the above 108 NSCLC patients, 75 were early stage NSCLC and 33 were advanced stage. For patients with early stage (I–II) NSCLC, the survival analysis demonstrated that high ezrin and ezrin^Thr-567^ levels were associated with lower overall survival rate (*P* = 0.016 and *P* = 0.045, respectively, log-rank) (Figure 4). However, ezrin^Tyr-353^ status was not correlated with the survival rate of patients with early stage NSCLC. Additionally, the expression statuses of ezrin, ezrin^Thr-567^ and ezrin^Tyr-353^ proteins were not correlated with the survival rate in patients with advanced stage (III–IV) NSCLC (data not shown, *P* = 0.104, *P* = 0.288, *P* = 0.713, respectively, log-rank).

## Discussion

The human ezrin gene maps to chromosome 6q25.2-q26 and the total length of the mRNA is 3166 bp, encoding 585 amino acids [6]. Ezrin is known to be a component of cell surface structures that are involved in cell adhesion to the extracellular matrix, as well as in cell-cell interactions, receptor tyrosine kinase signaling, signal transduction through Rho GTPase and interactions with Akt-mediated cellular apoptotic machinery [14,15]. It is present in the cytoplasm in an inactive form but after threonine and tyrosine phosphorylation, ezrin assumes an active form, moving to the cell membrane and tethering F-actin to the cell membrane [8]. Recent studies showed that ezrin likely regulates adhesion molecules and participates in cell signal transduction and other channels in these processes [6,16].

In previous studies, ezrin was found to have important roles in the tumorigenesis and metastasis of several malignancies. Khanna et al. [6] showed that high ezrin expression was associated with early development of metastasis and poor outcome in osteosarcoma. Xie et al. [17] reported that ezrin affected the growth and invasiveness of esophageal SCC cells through the MAPK and transforming growth factor β pathway. However, Moilanen et al. found that serous ovarian cancer with low expression of ezrin protein had poor prognosis [18]. Karmakar et al. [19] also reported that the invasion of choriocarcinoma cells actually strengthened when ezrin protein expression decreased. Here we also found that ezrin was significantly upregulated in NSCLC compared with the adjacent non-tumor tissues and normal lung tissue counterparts (P < 0.01). Ezrin overexpression was significantly correlated with the advanced clinical stage and poor differentiation of NSCLC (P < 0.01), but no correlation was found with lymph node metastasis and invasion of NSCLC in our data. However, Zhang et al. [20] reported that ezrin expression was significantly associated with increased tumor stage and LN metastasis of NSCLC. These studies suggested that there might be cell- and tissue-specific functions for ezrin in tumor progression.

To date, two important phosphorylation sites in ezrin have been identified: ezrin^Thr567^ and ezrin^Tyr353^. Ezrin directly interacts with signaling enzymes such as phosphoinositide 3-kinase (PI3-kinase), and the phosphorylated tyrosine 353 (Tyr-353) residue of ezrin is regulated through the PI3-kinase/Akt pathway [14]. Ezrin is also preferentially degraded and resynthesized after the phosphorylation at threonine 567 (Thr-567) depends on the p38 MAP-kinase activity [21]. Phosphorylation at Thr-567 has received a great deal of attention, as this phosphorylation event is believed to relieve the N- to C-terminal binding of ezrin, transforming ezrin into an active state with domains accessible for binding to membrane and F-actin [22]. This suggests that ezrin^Thr-567^ alters ezrin molecule plasticity, and it is associated with numerous biological behaviors. Chen et al. [23] revealed that ezrin^Thr-567^ has an important role in liver cancer metastasis. Krishnan et al. [24] found that transfection of the ezrin-Thr-567A mutant blocked the ezrin^Thr-567^-inhibited metastases in Ewing’s sarcoma, suggesting that ezrin^Thr-567^ is closely related to malignancy metastasis. Additionally, ezrin^Tyr-353^ has been known to be related to subunit p85, which activates the PI3K/Akt pathway and plays an important role in modulating tumor cell survival, invasion, and metastasis. Cui et al. [25] reported that expression of ezrin^Tyr-353^ correlated with less tumor differentiation and the presence of lymph node metastasis in pancreatic cancer. Lan et al. [26] found that induction of p-ezrin via the p38 MAP kinase signaling pathway was involved in the formation of microvilli during development of epithelial cell polarization. However, few reports to date have identified the favorable roles and prognostic value of p-ezrin in NSCLC. Zhang et al. [20] revealed that p-ezrin expression was significantly higher in NSCLC than in normal lung tissues, and was closely correlated with clinical stage and LN metastasis in NSCLC. In the present study, we found that p-ezrin exhibited cytosolic and membranous staining patterns in NSCLC, and the percentages of ezrin^Thr-567^ and ezrin^Tyr-353^ expression were significantly higher in NSCLC than in adjacent non-tumor tissues and normal tissue counterparts (*P* < 0.01). Interestingly, we found that total ezrin staining was lower than p-ezrin staining. Similarly, Oda et al. revealed that the expression rate of the ezrin was 42.5%, but 55.0% for ezrin^Tyr353^ and 42.5% for ezrin^Tyr354^ in intraductal papillary mucinous neoplasms of pancreas. Moreover, ezrin expression rate was 32.4%, but higher for ezrin^Tyr353^ (41.2%) in intestinal-type pancreatic neoplasms [27]. Cui et al. and Di Cristofano et al. also reported the similar conclusions in pancreatic cancer and osteosarcoma, respectively [25,28]. The cause might be related with that some cases mainly expressed p-ezrin, but showed lower ezrin level or not, suggesting that although total ezrin is sum of p-ezrin and non-phosphorylated ezrin in cell level, it is not suitable in the case study. It needs the further study to verify the detailed mechanism.

Among the clinicopathological features, both ezrin^Thr-567^ and ezrin^Tyr-353^ protein overexpression were significantly correlated with the poor differentiation and late clinical stage of NSCLC (*P* < 0.01). However, only ezrin^Thr-567^ overexpression was significantly correlated with the presence of lymph node metastasis, suggesting that ezrin^Thr-567^ was important for the invasion and metastasis processes in NSCLC. Interestingly, Orr et al. [29] recently found that EGF can induce ezrin phosphorylation (Thr567) via activation of the SK/S1P pathway, and Antelmi et al. [30] revealed that p-ezrin was almost exclusively expressed in invadopodia lipid rafts where it co-locolized in a functional complex with EGFR and β1-integrin in metastatic breast cancer cell line MDA-MB-231, suggesting that ezrin might be related with EGF and EGFR in cancer progression. These results impelled us to study the detailed mechanism of the correlation between EGFR and ezrin, and whether EGFR-TKI can suppress the metastasis of NSCLC *via* ezrin phosphorylation in future.

In regard to survival, Zhang et al. [20] showed that ezrin-positive expression independently predicted inferior overall survival and disease-free survival. Additionally, ezrin overexpression was helpful to predict the poor survival of patients with early stage of NSCLC. Cui et al. [25] also found that overall survival of patients with pancreatic cancer was significantly associated with ezrin^Tyr-353^, but not with total ezrin or ezrin^Thr-567^. However, Di Cristofano et al. [28] reported no statistical significance regarding the relationship between p-ezrin expression and survival time in osteosarcoma. Here we demonstrated that ezrin^Tyr-353^ has no correlation with the survival of patients with NSCLC. However, ezrin^Thr-567^ expression was significantly correlated with adverse outcomes with respect to overall survival time, and both ezrin and ezrin^Thr-567^ overexpressions were correlated with shorter survival time in patients with early stage NSCLC. The high proportion and prognostic value of ezrin and ezrin^Thr-567^ expression in NSCLC suggested that ezrin, especially ezrin^Thr-567^, could be a potential biomarker for NSCLC. However, more extensive investigations are needed to clarify the exact roles of ezrin and ezrin^Thr-567^ in the development and progression of NSCLC.

## Conclusions

Ezrin, ezrin^Thr-567^ and ezrin^Tyr-353^ were all significantly upregulated in NSCLC compared with normal tissues, and all correlated with the poor differentiation and late clinical stage of NSCLC. However, only ezrin^Thr-567^ overexpression correlated with the presence of lymph node metastasis of NSCLC. Additionally, ezrin^Thr-567^ correlated with the overall survival time of patients with NSCLC, and both ezrin and ezrin^Thr-567^ overexpression were correlated with shorter survival time in patients with early stage NSCLC. In this regard, ezrin, especially ezrin^Thr-567^, may prove to be useful as a novel prognostic biomarker of NSCLC.

## Competing interests

The authors declare that they have no competing interests.

## Authors’ contributions

JT, JJ, LX and SY participated in study conception, design, case selection and experiments. JT, SX and ZS carried out the data collection. JT, YK and LZ performed the scoring of immunohistochemical staining. JT, PY, SX and LZ performed data analysis and writing of the manuscript. All the authors read and approved the final manuscript.

## Pre-publication history

The pre-publication history for this paper can be accessed here:

http://www.biomedcentral.com/1471-2407/14/191/prepub

## References

[B1] Jemal A Siegel R Ward E Murray T Xu J Thun MJ Cancer statistics, 2007 CA Cancer J Clin 2007 57 43 66 10.3322/canjclin.57.1.43 17237035

[B2] Siegel R DeSantis C Virgo K Stein K Mariotto A Smith T Cooper D Gansler T Lerro C Fedewa S Lin C Leach C Cannady RS Cho H Scoppa S Hachey M Kirch R Jemal A Ward E Cancer treatment and survivorship statistics 2012 CA Cancer J Clin 2012 62 220 241 10.3322/caac.21149 22700443

[B3] Chen X Liu Y Røe OD Qian Y Guo R Zhu L Yin Y Shu Y Gefitinib or erlotinib as maintenance therapy in patients with advanced stage non-small cell lung cancer: a systematic review PLoS One 2013 8 3 e59314 10.1371/journal.pone.0059314 23555654 PMC3605444

[B4] Gaughan EM Costa DB Genotype-driven therapies for non-small cell lung cancer: focus on EGFR, KRAS and ALK gene abnormalities Ther Adv Med Oncol 2011 3 3 113 125 10.1177/1758834010397569 21904575 PMC3150063

[B5] Lee HW Kim EH Oh MH Clinicopathologic implication of ezrin expression in non-small cell lung cancer Korean J Pathol 2012 46 5 470 477 10.4132/KoreanJPathol.2012.46.5.470 23136574 PMC3490123

[B6] Khanna C Wan X Bose S Cassaday R Olomu O Mendoza A Yeung C Gorlick R Hewitt SM Helman LJ The membrane-cytoskeleton linker ezrin is necessary for osteosarcoma metastasis Nat Med 2004 10 2 182 186 10.1038/nm982 14704791

[B7] Zhu J Feng Y Ke Z Yang Z Zhou J Huang X Wang L Down-regulation of miR-183 promotes migration and invasion of osteosarcoma by targeting ezrin Am J Pathol 2012 180 6 2440 2451 10.1016/j.ajpath.2012.02.023 22525461

[B8] Bretscher A Edwards K Fehon RG ERM proteins and merlin: integrators at the cell cortex Nat Rev Mol Cell Biol 2002 3 586 599 10.1038/nrm882 12154370

[B9] Turunen O Wahlström T Vaheri A Ezrin has a COOH-terminal actin-binding site that is conserved in the ezrin protein family J Cell Biol 1994 126 1445 1453 10.1083/jcb.126.6.1445 8089177 PMC2290954

[B10] Liu Y Belkina NV Park C Nambiar R Loughhead SM Patino-Lopez G Ben-Aissa K Hao JJ Kruhlak MJ Qi H von Andrian UH Kehrl JH Tyska MJ Shaw S Constitutively active ezrin increases membrane tension, slows migration, and impedes endothelial transmigration of lymphocytes in vivo in mice Blood 2012 119 2 445 453 10.1182/blood-2011-07-368860 22106344 PMC3257010

[B11] Fievet BT Gautreau A Roy C Del Maestro L Mangeat P Louvard D Arpin M Phosphoinositide binding and phosphorylation act sequentially in the activation mechanism of ezrin J Cell Biol 2004 164 653 659 10.1083/jcb.200307032 14993232 PMC2172172

[B12] Elzagheid A Korkeila E Bendardaf R Buhmeida A Heikkilä S Vaheri A Syrjänen K Pyrhönen S Carpén O Intense cytoplasmic ezrin immunoreactivity predicts poor survival in colorectal cancer Hum Pathol 2008 39 12 1737 1743 10.1016/j.humpath.2008.04.020 18701134

[B13] Lin L Piao J Gao W Piao Y Jin G Ma Y Li J Lin Z DEK over expression as an independent biomarker for prognosis in colorectal cancers BMC Cancer 2013 13 366 10.1186/1471-2407-13-366 23902796 PMC3751154

[B14] Gautreau A Poullet P Louvard D Arpin M Ezrin, a plasma membrane-microfilament linker, signals cell survival through the phosphatidylinositol 3-kinase/Akt pathway Proc Natl Acad Sci U S A 1999 96 13 7300 7305 10.1073/pnas.96.13.7300 10377409 PMC22080

[B15] Martin GS Cell signaling and cancer Cancer Cell 2003 4 3 167 174 10.1016/S1535-6108(03)00216-2 14522250

[B16] McClatchey AI Merlin and ERM proteins: unappreciated roles in cancer development? Nat Rev Cancer 2003 3 11 877 883 10.1038/nrc1213 14668818

[B17] Xie JJ Xu LY Xie YM Zhang HH Cai WJ Zhou F Shen ZY Li EM Roles of ezrin in the growth and invasiveness of esophageal squamous carcinoma cells Int J Cancer 2009 124 11 2549 2558 10.1002/ijc.24216 19165868

[B18] Moilanen J Lassus H Leminen A Vaheri A Bützow R Carpén O Ezrin immunoreactivity in relation to survival in serous ovarian carcinoma patients Gynecol Oncol 2003 90 2 273 281 10.1016/S0090-8258(03)00262-2 12893187

[B19] Karmakar S Das C Modulation of ezrin and E-cadherin expression by IL-1beta and TGF-beta1 in human trophoblasts J Reprod Immunol 2004 64 1–2 9 29 15596224 10.1016/j.jri.2004.04.005

[B20] Zhang XQ Chen GP Wu T Yan JP Zhou JY Expression and clinical significance of ezrin in non-small cell lung cancer Clin Lung Cancer 2012 13 3 196 204 10.1016/j.cllc.2011.04.002 22137559

[B21] Grune T Reinheckel T North JA Li R Bescos PB Shringarpure R Davies KJ Ezrin turnover and cell shape changes catalyzed by proteasome in oxidatively stressed cells FASEB J 2002 16 12 1602 1610 10.1096/fj.02-0015com 12374783

[B22] Zhu L Zhou R Mettler S Wu T Abbas A Delaney J Forte JG High turnover of ezrin Thr567 phosphorylation: conformation, activity, and cellular function Am J Physiol Cell Physiol 2007 293 3 874 884 10.1152/ajpcell.00111.2007 17553936

[B23] Chen Y Wang D Guo Z Zhao J Wu B Deng H Zhou T Xiang H Gao F Yu X Liao J Ward T Xia P Emenari C Ding X Thompson W Ma K Zhu J Aikhionbare F Dou K Cheng SY Yao X Rho kinase phosphorylation promotes ezrin-mediated metastasis in hepatocellular carcinoma Cancer Res 2011 71 1721 1729 10.1158/0008-5472.CAN-09-4683 21363921 PMC3119000

[B24] Krishnan K Bruce B Hewitt S Thomas D Khanna C Helman LJ Ezrin mediates growth and survival in Ewing’s sarcoma through the AKT/mTOR, but not the MAPK, signaling pathway Clin Exp Metastasis 2006 23 227 236 10.1007/s10585-006-9033-y 17028919

[B25] Cui Y Li T Zhang D Han J Expression of ezrin and phosphorylated ezrin (pEzrin) in pancreatic ductal adenocarcinoma Cancer Invest 2010 28 3 242 247 10.3109/07357900903124498 20158339

[B26] Lan M Kojima T Murata M Osanai M Takano K Chiba H Sawada N Phosphorylation of ezrin enhances microvillus length via a p38 MAP-kinase pathway in an immortalized mouse hepatic cell line Exp Cell Res 2006 312 2 111 120 10.1016/j.yexcr.2005.09.018 16274688

[B27] Oda Y Aishima S Morimatsu K Hayashi A Shindo K Fujino M Mizuuchi Y Hattori M Tanaka M Oda Y Differential ezrin and phosphorylated ezrin expression profiles between pancreatic intraepithelial neoplasia, intraductal papillary mucinous neoplasm, and invasive ductal carcinoma of the pancreas Hum Pathol 2013 44 8 1487 1498 10.1016/j.humpath.2012.12.001 23465281

[B28] Di Cristofano C Leopizzi M Miraglia A Sardella B Moretti V Ferrara A Petrozza V Della Rocca C Phosphorylated ezrin is located in the nucleus of the osteosarcoma cell Mod Pathol 2010 23 7 1012 1020 10.1038/modpathol.2010.77 20348881

[B29] Orr Gandy KA Adada M Canals D Carroll B Roddy P Hannun YA Obeid LM Epidermal growth factor-induced cellular invasion requires sphingosine-1-phosphate/sphingosine-1-phosphate 2 receptor-mediated ezrin activation FASEB J 2013 27 8 3155 3166 10.1096/fj.13-228460 23629860 PMC3714586

[B30] Antelmi E Cardone RA Greco MR Rubino R Di Sole F Martino NA Casavola V Carcangiu M Moro L Reshkin SJ ß1 integrin binding phosphorylates ezrin at t567 to activate a lipid raft signalsome driving invadopodia activity and invasion PLoS One 2013 8 9 e75113 10.1371/journal.pone.0075113 24086451 PMC3782503

